# Venetoclax, a BCL-2 Inhibitor, Enhances the Efficacy of Chemotherapeutic Agents in Wild-Type ABCG2-Overexpression-Mediated MDR Cancer Cells

**DOI:** 10.3390/cancers12020466

**Published:** 2020-02-18

**Authors:** Jing-Quan Wang, Jonathan Y. Li, Qiu-Xu Teng, Zi-Ning Lei, Ning Ji, Qingbin Cui, Leli Zeng, Yihang Pan, Dong-Hua Yang, Zhe-Sheng Chen

**Affiliations:** 1 Department of Pharmaceutical Sciences, College of Pharmacy and Health Sciences, St. John’s University, Queens, NY 11439, USA; jingquan.wang16@stjohns.edu (J.-Q.W.); jonathanli173@gmail.com (J.Y.L.); qiuxu.teng15@stjohns.edu (Q.-X.T.); zining.lei14@stjohns.edu (Z.-N.L.); jining@ihcams.ac.cn (N.J.); qingbc@gmail.com (Q.C.); zengleli0736@163.com (L.Z.); 2 School of Public Health, Guangzhou Medical University, Guangzhou 511436, China; 3 Precision Medicine Center, Research Center, The Seventh Affiliated Hospital, Sun Yat-sen University, Shenzhen 518107, China; yihang73@hotmail.com

**Keywords:** venetoclax, ABCG2, MDR, chemotherapy, ABC transporter

## Abstract

Previous studies have shown that small-molecule BCL-2 inhibitors can have a synergistic interaction with ABCG2 substrates in chemotherapy. Venetoclax is a potent and selective BCL-2 inhibitor, approved by the FDA in 2016 for the treatment of patients with chronic lymphocytic leukemia (CLL). This study showed that, at a non-toxic concentration, venetoclax at 10 µM significantly reversed multidrug resistance (MDR) mediated by wild-type ABCG2, without significantly affecting MDR mediated by mutated ABCG2 (R482G and R482T) and ABCB1, while moderate or no reversal effects were observed at lower concentrations (0.5 to 1 µM). The results showed that venetoclax increased the intracellular accumulation of chemotherapeutic agents, which was the result of directly blocking the wild-type ABCG2 efflux function and inhibiting the ATPase activity of ABCG2. Our study demonstrated that venetoclax potentiates the efficacy of wild-type ABCG2 substrate drugs. These findings may provide useful guidance in combination therapy against wild-type ABCG2-mediated MDR cancer in clinical practice.

## 1. Introduction

The incidence of death caused by cancer has increased worldwide. According to the annual cancer report released by the World Health Organization (WHO), there were around 18.1 million new cases and 9.6 million cancer deaths worldwide in 2018 [1]. Currently, chemotherapy is a major strategy in treatment against various types of cancers [2]. However, multi-drug resistance (MDR) remains one of the leading obstacles in cancer chemotherapy [3,4,5]. Cancer cells with MDR becomes cross-resistant to structurally unrelated chemotherapeutic drugs. Numerous cellular and non-cellular pathways have been proposed as theoretical mechanisms behind MDR [6], including poor vasculatures [7], increased enzyme level of xenobiotic metabolism (e.g., glutathione-S-transferase) [8] and transport-based mechanisms [4,9]. The transport-based MDR involves the efflux of drug by ATP-dependent membrane transporters, which limit the therapeutic drug concentration of cancer cells [6].

ATP-binding cassette (ABC) transporters are a superfamily of membrane proteins mediating cancer MDR in multiple types of cancers [10,11]. An important member of this superfamily is ABC transporter subfamily G member 2 (ABCG2), which is also named the breast cancer resistance protein (BCRP). Elevated ABCG2 expression in cancer cells results in resistance to a wide spectrum of chemotherapeutic agents, including mitoxantrone, topotecan, SN-38 and doxorubicin [12,13]. Numerous studies have revealed that some tumors that are refractory to anticancer drugs are also those with high levels of ABCG2 expression [14]. Therefore, it will be beneficial to find out novel inhibitors of ABCG2 and understand the mechanism of their MDR modulation.

**Figure 1:**
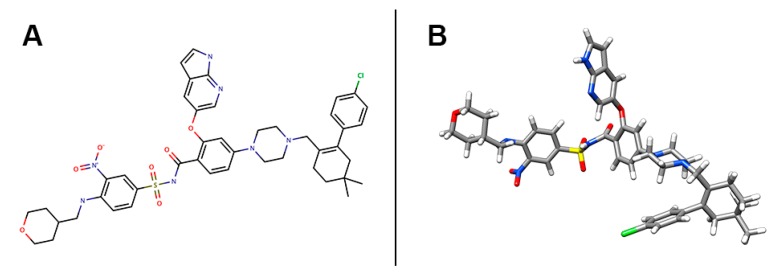
Chemical structure of venetoclax. (**A**) 2-D view of venetoclax structure. (**B**) 3-D view of venetoclax structure. Venetoclax molecule was displayed as colored sticks. Grey: carbon, blue: nitrogen, red: oxygen, yellow: sulfur, while: hydrogen, green: chloride.

Previous studies have shown that small-molecule BCL-2 inhibitors can have a synergistic interaction with ABCG2 substrates, including flavopiridol [15,16] and benzimidazoles [17,18], while few mentioned the mechanism related to the alteration of the ABCG2 function. There has been no report on the direct interaction between BCL-2 inhibitors and a specific ABC transporter such as ABCG2. In this study, we studied venetoclax (Venclexta, Venclyxto, ABT-199, GDC-0199, RG7601), a potent and selective BCL-2 inhibitor, approved by the FDA in 2016 for the treatment of patients with chronic lymphocytic leukemia (CLL) with 17p deletion [19]. The chemical structure of venetoclax is shown in Figure 1. Venetoclax has been reported to enhance treatment response in combination therapy with ABCG2 substrates including tamoxifen [20,21] and ibrutinib [22]. Additionally, a previous study provided evidence that venetoclax was a potential ABCG2 inhibitor, but a further mechanism was not reported [23]. We found that venetoclax enhanced the efficacy of ABCG2-substrate anticancer agents by directly inhibiting ABCG2-ATPase activity, blocking the efflux function of wild-type ABCG2, and therefore, increasing the intracellular accumulation of chemotherapeutic drugs. Intermolecular interaction calculated by a docking simulation suggested that venetoclax could bind to the drug-binding pocket and ATP-binding site of ABCG2. This study revealed a potential mechanism of the synergistic effect of venetoclax with certain chemotherapeutic agents, thus indicating that venetoclax may be beneficial in combination treatments of MDR cancers.

## 2. Results

### 2.1. Venetoclax Showed Similar Cytotoxicity in both Parental and Resistant Cell Lines

**Figure 2:**
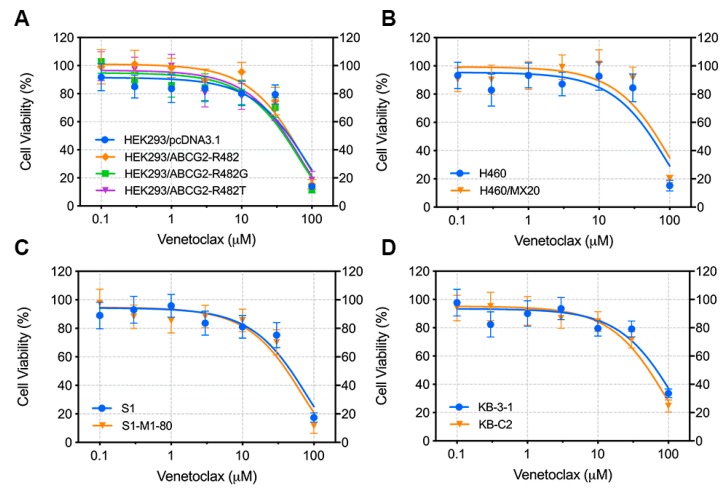
Cytotoxicity of venetoclax in different cell lines. (**A**) Viability-concentration curve for HEK293 transfected cell lines (HEK293/pcDNA3.1, HEK293/ABCG2-R482, HEK293/ABCG2-R482G, HEK293/ABCG2-R482T) with gradient concentrations of venetoclax (0–100 µM). (**B**) Viability-concentration curve for NCI-H460 and ABCG2/R482-overexpressing NCI-H460/MX20 with gradient concentrations of venetoclax (0–100 µM). (**C**) Viability-concentration curve for S1 and ABCG2/R482G-overexpressing S1-M1-80 with gradient concentrations of venetoclax (0–100 µM). (**D**) Viability-concentration curve for KB-3-1 and ABCB1-overexpressing KB-C2 with gradient concentrations of venetoclax (0–100 µM). Curves were generated by exponential nonlinear regression fit. Points with error bars represent mean ± SD from 3 independent triplicate experiments.

We first evaluated the cytotoxicity of venetoclax on different cell lines, where cells were cultured with gradient concentrations of venetoclax for 72 h and cell viability was measured by the MTT assay. Cell lines include HEK293/pcDNA3.1 + HEK293/ABCG2-R482 + HEK293/ABCG2-R482G + HEK293/ABCG2-R482T (vector control and ABCG2-transfected cell lines), H460 + H460/MX20 (parental and drug-selected wild-type overexpression cell lines), S1 + S1-M1-80 (parental and drug-selected R482G ABCG2 overexpression cell lines), and KB-3-1 + KB-C2 (parental and drug-selected ABCB1 overexpressing cell line). We found that venetoclax showed similar IC_50_ values in different pairs of parental/resistant cell lines, which indicates that venetoclax is not a potential substrate of ABCG2 or ABCB1(Figure 2). Moreover, a safe concentration (10 µM) of venetoclax, without causing significant cell death (viability > 80%), was chosen based on the cytotoxicity assay for the following reversal studies.

### 2.2. Venetoclax Re-Sensitized Transfected Wild-Type ABCG2-Overexpressing MDR Cells without Affecting Mutated ABCG2-Overexpressing and ABCB1-Overexpressing MDR Cells

**Figure 3:**
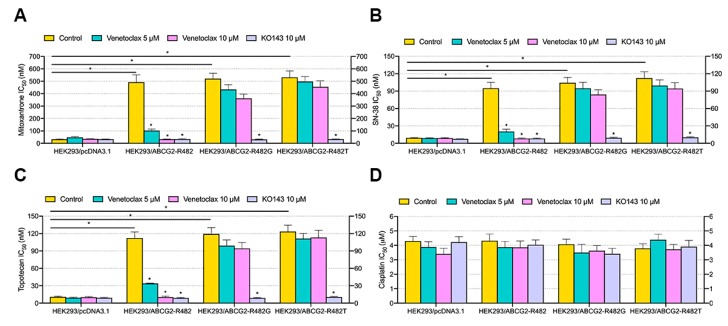
Effect of venetoclax on MDR in transfected ABCG2-overexpressing cells. (**A**) IC_50_ of mitoxantrone in HEK293 transfected cells with/without venetoclax and KO143. (**B**) IC_50_ of SN-38 in HEK293 transfected cells with/without venetoclax and KO143. (**C**) IC_50_ of topotecan in HEK293 transfected cells with/without venetoclax and KO143. (**D**) IC_50_ of cisplatin in HEK293 transfected cells with/without venetoclax and KO143. Columns with error bars represent mean ± SD from 3 independent triplicate experiments. Asterisks (*) indicate *p* < 0.05 versus control group (HEK293/pcDNA3.1 or untreated resistant cells).

Two drug concentrations (5 and 10 µM) of venetoclax were selected for the reversal study. First, venetoclax and chemotherapeutic agents (mitoxantrone, topotecan and SN-38) were added and cultured with a set of single-factor transfected ABCG2-overexpressing MDR HEK293 cells. According to Figure 3, IC_50_ values of mitoxantrone (Figure 3A), SN-38 (Figure 3B) and topotecan (Figure 3C) were significantly decreased with the addition of 5 or 10 µM venetoclax in HEK293/ABCG2-R482 cells, compared with the control cells. Moreover, such antagonist effect is comparable to that of the positive modulator KO143 at the same concentration (10 µM). In mutant ABCG2-overexpressing (R482G and R482T) cells, venetoclax did not show a statistically significant reversal effect against corresponding MDR. The IC_50_ of cisplatin, a non-substrate of ABCG2 transporter, was not affected by the addition of either venetoclax or KO143. These results indicate that venetoclax is a potent reversal agent specifically against wild type ABCG2-mediated MDR.

### 2.3. Venetoclax Re-Sensitized Drug-Selected Wild Type ABCG2-Overexpressing MDR Cancer Cells without Affecting Drug-Selected Mutated ABCG2-Overexpressing and ABCB1-Overexpressing Cancer Cells

**Figure 4:**
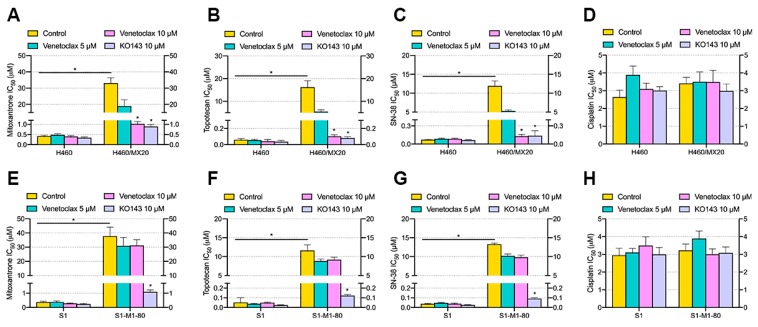
Effect of venetoclax on MDR in drug-selected ABCG2-overexpressing cells. (**A**) IC_50_ of mitoxantrone in NCI-H460 and NCI-H460/MX20 with/without venetoclax and KO143. (**B**) IC_50_ of topotecan in NCI-H460 and NCI-H460/MX20 with/without venetoclax and KO143. (**C**) IC_50_ of SN-38 in NCI-H460 and NCI-H460/MX20 with/without venetoclax and KO143. (**D**) IC_50_ of cisplatin in NCI-H460 and NCI-H460/MX20 with/without venetoclax and KO143. (**E**) IC_50_ of mitoxantrone in S1 and S1-M1-80 with/without venetoclax and KO143. (**F**) IC_50_ of topotecan in S1 and S1-M1-80 with/without venetoclax and KO143. (**G**) IC_50_ of SN-38 in S1 and S1-M1-80 with/without venetoclax and KO143. (**H**) IC_50_ of cisplatin in S1 and S1-M1-80 with/without venetoclax and KO143. Columns with error bars represent mean ± SD from 3 independent triplicate experiments. Asterisks (*) indicate *p* < 0.05 versus parental group (H460 or S1).

To further explore the reversal effect of venetoclax, two drug-selected MDR cancer and respective parental cell lines were used. Specifically, NCI-H460/MX20 overexpresses wild-type ABCG2 and S1-M1-80 overexpresses G-mutant ABCG2, verified by DNA sequencing [24]. As shown in Figure 4, drug-selected MDR cells (NCI-H460/MX20 and S1-M1-80) showed significant resistance, compared with parental cells (NCI-H460 and S1). For the reversal effect, venetoclax significantly lowered the IC_50_ values of mitoxantrone, topotecan and SN-38 at 10 µM in NCI-H460/MX20 cells compared with the untreated group, while in S1-M1-80 cells, the IC_50_ values of three anticancer drugs were not significantly altered by venetoclax at both concentrations. The IC_50_ values of cisplatin were not altered. These results were consistent with the findings in Section 2.2 showing that venetoclax re-sensitized wild type ABCG2-mediated MDR cells without affecting MDR mediated by mutated ABCG2. Moderate reversal effects of venetoclax were observed at lower concentrations (500 nM to 1 µM, Figure S1). The mechanism of the reversal effect was explored in following sections.

### 2.4. Venetoclax Increased the Intracellular Accumulation of Mitoxantrone[^3^H] in Wild Type ABCG2-Overexpressing Cells but not in Mutated ABCG2-Overexpressing Cells

**Figure 5:**
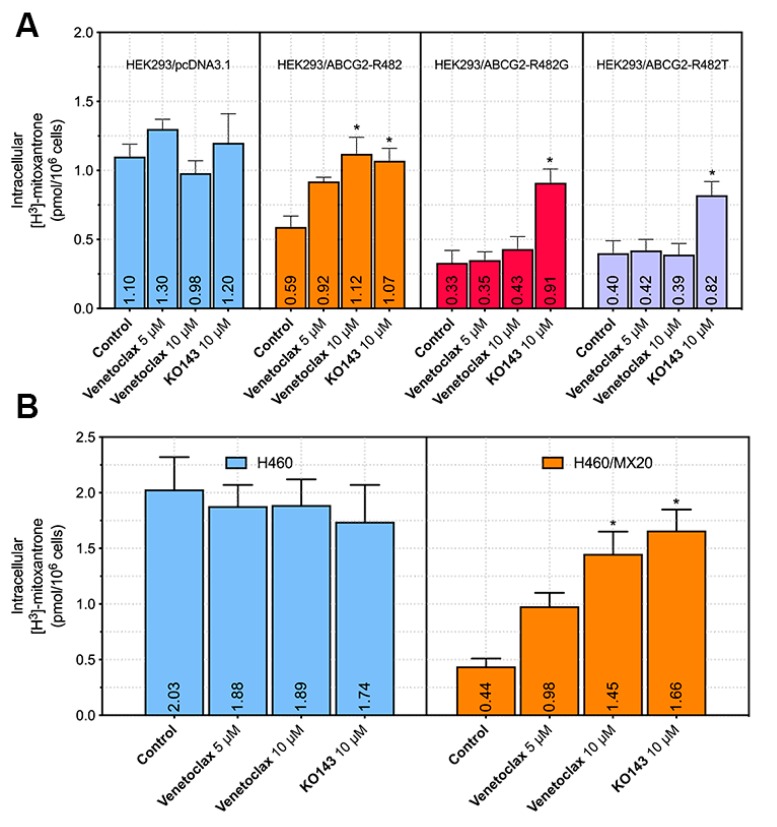

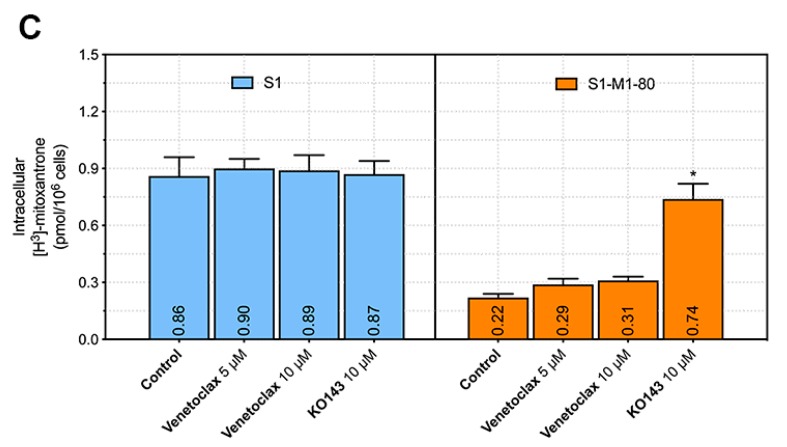
Effect of venetoclax on the intracellular accumulation of mitoxantrone[^3^H] in parental and ABCG2-overexpressing cells. (**A**) Effect of venetoclax on mitoxantrone[^3^H] accumulation in HEK293/pcDNA3.1, HEK293/ABCG2-R482, HEK293/ABCG2-R482G and HEK293/ABCG2-R482T cells. (**B**) Effect of venetoclax on mitoxantrone[^3^H] accumulation in NCI-H460 and NCI-H460/MX20 cells. (**C**) Effect of venetoclax on mitoxantrone[^3^H] accumulation in S1 and S1-M1-80 cells. Columns with error bars represent mean ± SD from 3 independent duplicate experiments. Asterisks (*) indicate *p* < 0.05 versus control group (untreated resistant cells).

The above results demonstrated the reversal effect of venetoclax in wild type ABCG2-overexpressing MDR cells. To obtain more insight into the mechanism of action, intracellular ABCG2 substrate accumulation was measured in different ABCG2-mediated MDR cell lines. As shown in Figure 5A, venetoclax significantly increased the intracellular accumulation of mitoxantrone[^3^H] in HEK293/ABCG2-R482 cells, while it did not significantly change its level in HEK293/ABCG2-R482G or HEK293/ABCG2-R482T cells. It is worth noting that the elevation of intracellular accumulation in HEK293/ABCG2-R482 cells by venetoclax is comparable to that of the positive modulator KO143, which indicated that venetoclax is an efficient modulator of wild-type ABCG2 with high specificity. In drug-selected ABCG2-overexpressing cells (Figure 5B,C), venetoclax at a higher concentration (10 µM) also significantly increased mitoxantrone[^3^H] accumulation in NCI-H460/MX20 cells, which overexpresses wild-type ABCG2. However, in ABCG2-R482G-overexpressing S1-M1-80 cells, intracellular accumulation of mitoxantrone[^3^H] was not significantly increased. These results indicated that one possible mechanism of venetoclax reversing MDR was via increasing the intracellular accumulation of anticancer agents, which leads to cell death.

### 2.5. Venetoclax Inhibited the Efflux of Mitoxantrone[^3^H] in Wild-Type ABCG2-Overexpressing Cells but not in Mutated ABCG2-Overexpressing Cells

**Figure 6:**
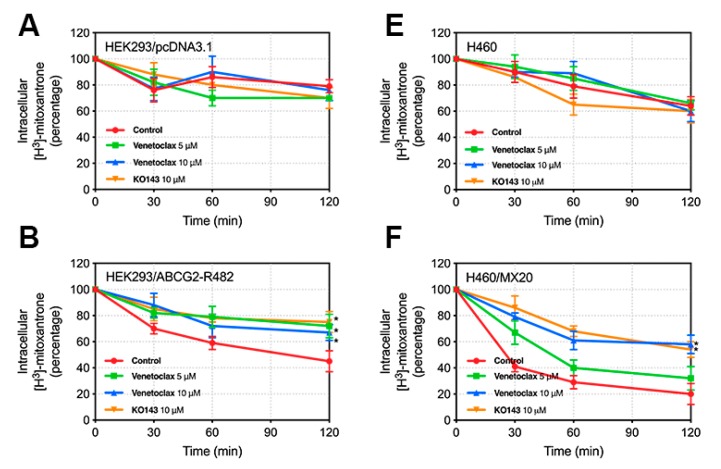

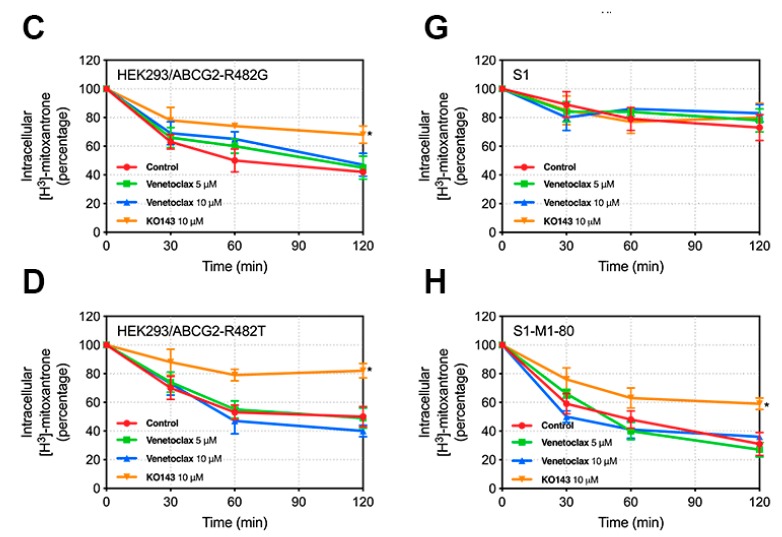
Effect of venetoclax on the efflux of mitoxantrone[^3^H] in parental and ABCG2-overexpressing cells. Time course (0/30/60/120 min) versus the percentage of remaining intracellular mitoxantrone[^3^H] was plotted to illustrate the effect of venetoclax on mitoxantrone[^3^H] efflux in (**A**) HEK293/pcDNA3.1, (**B**) HEK293/ABCG2-R482, (**C**) HEK293/ABCG2-R482G, (**D**) HEK293/ABCG2-R482T, (**E**) NCI-H460, (**F**) NCI-H460/MX20, (**G**) S1 and (**H**) S1-M1-80 cells. Points with error bars represent mean ± SD from 3 independent duplicate experiments. Asterisks (*) indicate *p* < 0.05 versus control group (untreated resistant cells).

To further confirm whether the elevated intracellular accumulation of mitoxantrone was due to the blockage of the ABCG2 efflux function, we observed the efflux of mitoxantrone[^3^H] in parental and resistant cell lines. In Figure 6, the remaining intracellular amount of mitoxantrone[^3^H] in ABCG2-overexpresing cells was significantly lower than that of HEK293/pcDNA3.1, NCI-H460 and S1 cells in the absence of venetoclax (Figure 6B–H). Treatment with 10 µM venetoclax significantly decreased the efflux of mitoxantrone[^3^H] in all wild-type ABCG2-overexpressing cells (HEK293/ABCG2-R482 and NCI-H460/MX20) in a time-dependent manner, while not significantly affecting the mutated ABCG2-overexpressing cells (HEK293/ABCG2-R482G, HEK293/ABCG2-R482T and S1-M1-80). Furthermore, the results in HEK293/ABCG2-R482 and NCI-H460/MX20 were comparable to that of the positive control KO143 at 10 µM.

### 2.6. Venetoclax Did Not Affect the Protein Expression of Wild-Type or Mutated ABCG2

**Figure 7:**
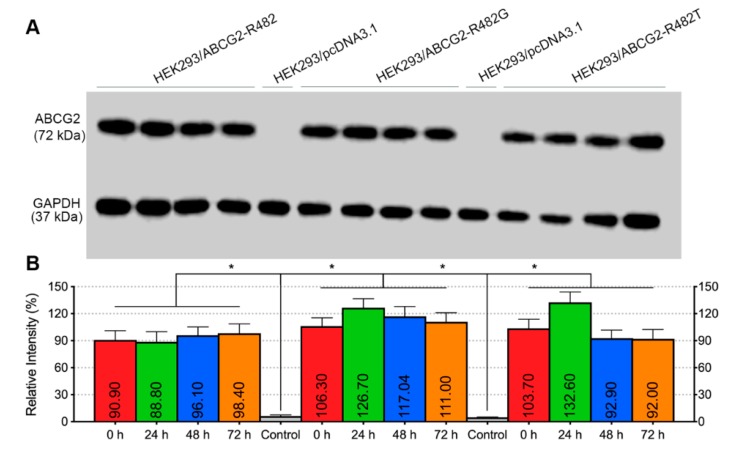

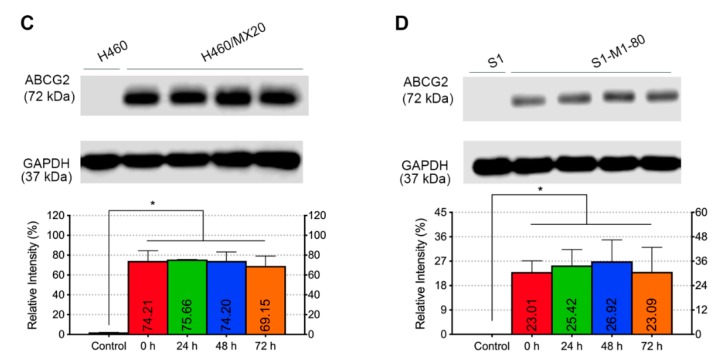
Effect of venetoclax on the ABCG2 protein expression. (**A**) ABCG2 protein expression after incubation with 10 µM venetoclax at different time points in HEK293/pcDNA3.1, HEK293/ABCG2-R482, HEK293/ABCG2-R482G and HEK293/ABCG2-R482T cells. GAPDH was used as a loading control. (**B**) Expression level quantification by grey scale values. (**C**) ABCG2 protein expression after incubation with 10 µM venetoclax at different time points in H460 and H460/MX20 cells. (**D**) ABCG2 protein expression after incubation with 10 µM venetoclax at different time points in S1 and S1-M1-80 cells. Columns with error bars represent mean ± SD from 3 independent triplicate experiments. Asterisks (*) indicate *p* < 0.05 versus parental group (HEK293/pcDNA3.1).

Another possible mechanism of the reversal effect is the down-regulation of the transporter expression level. To verify this, we performed Western blotting to evaluate ABCG2 protein expression in MDR cells. The results in Figure 7A,B indicated that treatment with venetoclax at various time points ranging from 0 to 72 h had no effect on the protein expression of either wild type (lane 1–4, from left to right) or mutated ABCG2 (R482G, lane 6–9; R482T, lane 11–14). Moreover, Figure 7C,D showed the results in drug-selected cancer cell lines H460 + H460/MX20 and S1 + S1-M1-80 (full blotting in Figures S2–S4). No significant alteration was observed after incubation with venetoclax for different periods of time. These results showed that the ABCG2-mediated MDR reversal was not due to the down-regulation of protein expression.

### 2.7. Venetoclax Inhibited the ATPase Activity of ABCG2

**Figure 8:**
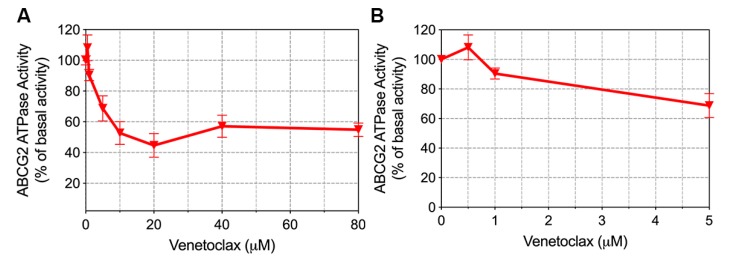
Effect of venetoclax on the vanadate-sensitive ABCG2 ATPase activity. (**A**) Concentration of venetoclax (0–80 µM) versus ATPase activity (percentage of basal activity) was plotted. (**B**) Concentration of venetoclax (0–5 µM) versus ATPase activity (percentage of basal activity) was plotted. Points with error bars represent mean ± SD from 3 independent duplicate experiments.

The ABCG2-mediated ATP hydrolysis was measured to determine the effect of venetoclax on wild-type ABCG2 ATPase activity. ABCG2-overexpressing cell membrane was treated with gradient concentrations of venetoclax. From the results in Figure 8, venetoclax inhibited vanadate-sensitive ATPase activity in a concentration-dependent manner and the ATPase activity reached a plateau of 44%. The inhibitory effect reached half-maximal response at a concentration of 4.78 µM. This finding clearly indicates the ability of venetoclax to interact with ABCG2 as an ATPase inhibitor.

### 2.8. Molecular Docking Simulation

**Figure 9:**
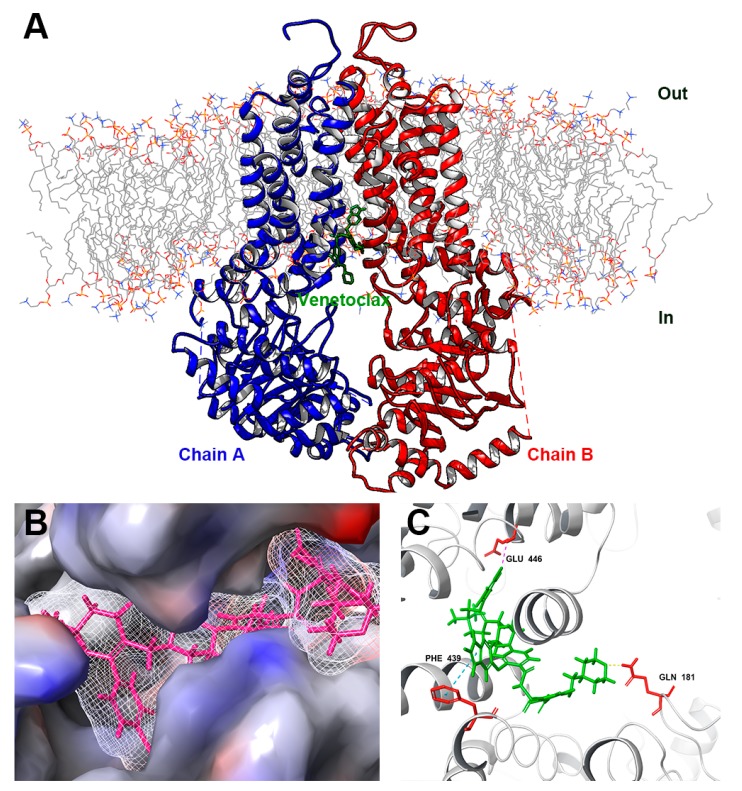
Interaction between venetoclax and ABCG2 protein by docking simulation. (**A**) An overview of top-scored docked position. Venetoclax was colored green. Two chains showed as ribbons were colored red and blue. Lipid molecules in cytoplasm membrane were depicted as colored lines. (**B**) Interaction between venetoclax and ABCG2 binding pocket with molecular surface displayed. (**C**) Docked conformation of venetoclax and wild-type ABCG2. Venetoclax was colored green and important residues were colored red. π-π stacking was represented as cyan dotted line, hydrogen bond was represented as yellow dotted line, halogen bond was represented as magenta dotted line.

To further understand the interaction of venetoclax with wild-type ABCG2 protein, a docking simulation was conducted. The best affinity score (−12.1 kcal/mol) showed that venetoclax may bind to the inhibitor binding site of the ABCG2 protein. According to the docked conformation shown in Figure 9, the pyrrole ring of venetoclax has π-π stacking interaction with the phenol ring of PHE 439 of ABCG2. The tetrahydropyran of venetoclax formed a hydrogen bond with the amine group of GLN 181 of ABCG2. Venetoclax was also stabilized by forming a halogen bond at the chloride group with GLU 446 of ABCG2. In addition, venetoclax can have hydrophobic interactions with multiple residues of ABCG2, such as THR 538, PHE 439, VAL 442, SER 443, VAL 445, LEU 539 and THR 538, which formed a ligand-binding pocket and stabilized the binding of venetoclax with ABCG2.

### 2.9. Molecular Dynamics Simulation

**Figure 10:**
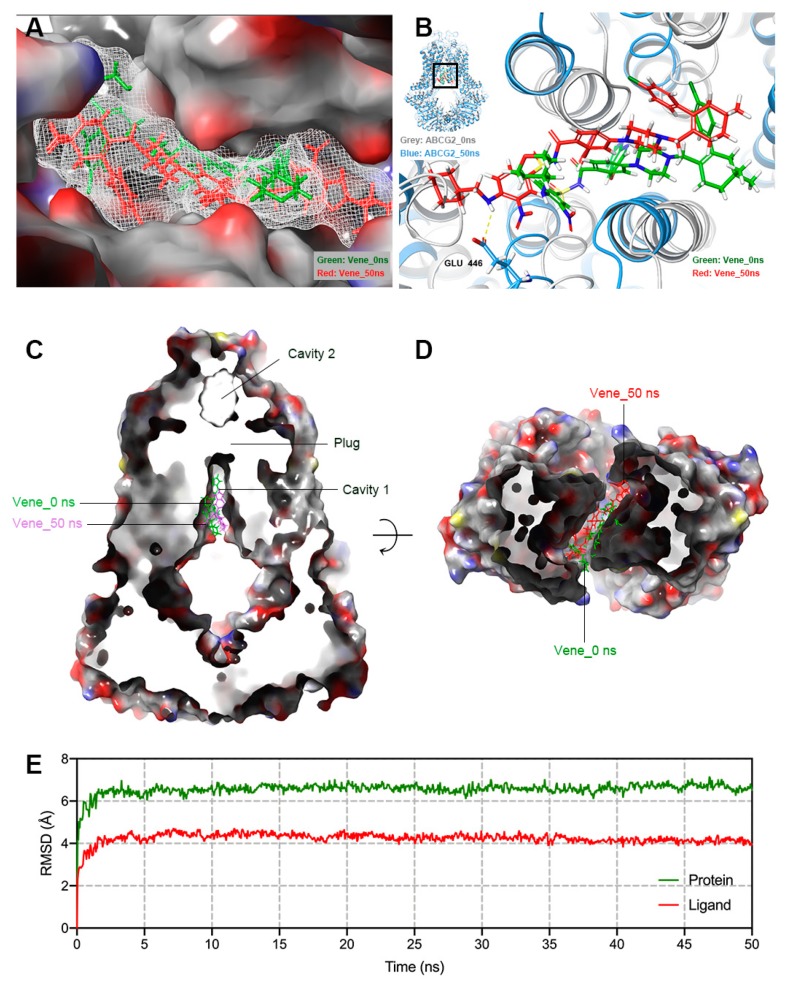
Molecular dynamics simulation (50 ns) of venetoclax-ABCG2 complex. (**A**) Position change of venetoclax in ABCG2 binding pocket before (green) and after (red) 50 ns MD simulation. Protein surface was displayed in colored (by element) solid style. (**B**) Potential chemical bonds found in post-MD conformation of venetoclax-ABCG2 complex. (**C**) Vertical slice through a surface representation of post-MD ABCG2, with bound venetoclax shown as colored sticks (green: pre-MD, red: post-MD). Leucine plug, cavity 1 and 2 were also indicated. (**D**) Cavity 1 viewed from a horizon slice at the position of cytoplasm membrane and bound venetoclax shown as colored sticks (green: pre-MD, red: post-MD). (**E**) Root mean square deviation (RMSD) of ABCG2 (green) and venetoclax (red) versus reference time (ns).

To further evaluate the binding conformation and stability, we conducted a 50 ns MD simulation using the highest-scoring result generated in Section 2.8. According to Figure 10E, both the ligand and protein (backbone) were stabilized and reached equilibrium after the first 5 ns, and remained stable until the end of the simulation. Venetoclax (ligand) had a displacement of 4 Å and ABCG2 (protein) was stable with a root mean square deviation (RMSD) of around 6 Å, indicating only small internal fluctuation inside the drug-binding site of the protein. Binding details before and after simulation are displayed in Figure 10B. After 50 ns, venetoclax remained in the same binding pocket and was likely to form a hydrogen bond at the amine group with GLU 446 (Figure 10A,B); moreover, venetoclax was bound in the cavity 1 of inward-facing ABCG2 (Figure 10C,D).

## 3. Discussion

In this study, we validated the antagonist effect of venetoclax in ABCG2-overexpressing MDR cells, which could provide valuable information on potent, developing combination treatments for MDR cancers. Cancers with MDR protect themselves from structurally-unrelated chemotherapeutic agents; thus, MDR is one of the major reasons for the failure of chemotherapy [3,25]. ABC transporters play a key role in cancer MDR, and studies on overcoming chemoresistance by modulating ABC transporters activity have always been important in cancer research [14,26,27]. ABCG2, as an important member of the ABC transporter superfamily, has been thoroughly studied since its first isolation from drug-resistant human breast cancer cell lines [28]. ABCG2 is a 72 kDa, 655-amino acid protein composed of six transmembrane domains including an N-terminal ATP-binding domain and C-terminal transmembrane domain [12]. As a “half-transporter”, ABCG2 most likely exists as homodimers [29]. ABCG2 was discovered in many organs in humans, including liver, kidney, GI tract, CNS, testes, ovaries, adrenal glands and placenta [30]. Substrates of ABCG2 span across different classes, including, but not limited to, topoisomerase inhibitors (mitoxantrone, topotecan, SN-38), antimetabolites (methotrexate), tyrosine kinase inhibitors (imatinib, gefitinib) and non-chemotherapeutic drugs (cimetidine, sulfasalazine, rosuvastatin) [31,32,33,34,35,36,37]. The FDA has approved clinical trials of combinational treatments using a BCL-2 inhibitor and other chemotherapeutic agents, such as venetoclax + ibrutinib (phase I/II, NCT02427451) and venetoclax + idarubicin (phase I/II, NCT03214562). As a BCL-2 inhibitor, venetoclax functions by inhibiting anti-apoptotic BCL-2 protein to induce apoptosis [38]. Meanwhile, it also functions as an ABCG2 modulator, as found in this study, by directly interacting with the ABCG2 protein and increasing ABCG2-substrate chemotherapeutic drugs efficacy.

**Figure 11:**
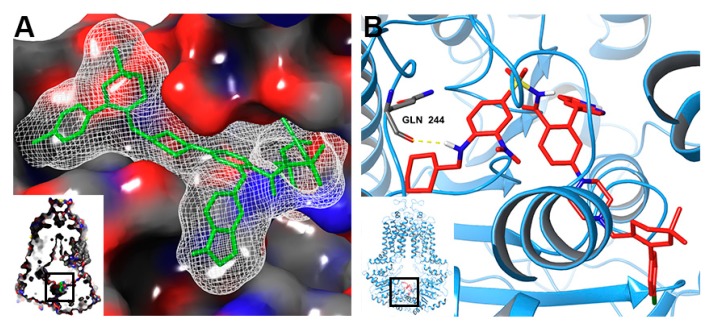
Docked conformation of venetoclax within the ATP-binding site of wild-type ABCG2. (**A**) Interaction between venetoclax and the ATP-binding domain of ABCG2 with molecular surface displayed. Venetoclax was displayed as green sticks. (**B**) Docked conformation of venetoclax and the ATP-binding domain of ABCG2. Venetoclax and important residues were displayed as colored sticks. Hydrogen bond was represented as a yellow dotted line.

Our study demonstrated that venetoclax at a relatively high concentration (10 µM) efficiently reversed MDR mediated by wild-type ABCG2 without significantly affecting MDR cells overexpressing R482G and R482T mutants of ABCG2, while lower concentrations (0.5–1.0 µM) of venetoclax only showed moderate reversal effects. According to the proposed transport mechanism, substrates bind to cavity 1 and ATP binds to the ATP-binding site. With the hydrolysis of ATP, ABCG2 transforms from inward-facing to outward-facing conformation, while the leucine plug opens and the substrate is released through the fused cavity formed by cavity 1 and 2 [39]. Thus, competitive binding of venetoclax on cavity 1 could inhibit the efflux and increase the intracellular accumulation of substrates. Inhibition of ATPase activity indicated that venetoclax might also interact with the ATP-binding site of wild type ABCG2. An extra docking simulation on the ATP-binding site predicted a high score (−9.9 kcal/mol), as shown in Figure 11, which indicated that venetoclax bound and stabilized with both cavity 1 and the ATP-binding site.

Drug-induced mutations and genetic polymorphism could change the arginine at position 482 of ABCG2 to a threonine (R > T) or glycine (R > G), causing altered transportation of substrates such as anthracycline and rhodamine 123 [40,41,42]. Additionally, previous studies reported altered ABCG2 modulator efficacy in different ABCG2 mutants including novobiocin (selectively inhibits wild-type ABCG2) and AC220 (selectively inhibits wild-type and R482T ABCG2) [43,44]. Amino acid residue 482 in ABCG2 plays important roles in protein functions, but the chemical nature of this side chain, crucially, does not determine the interaction between substrates and ABCG2 [24]. A previous study on R482X mutants revealed that residue 482 is likely indirectly involved in substrate binding by participating in the enhancement of conformational changes and/or intramolecular cross-talk which conveys the signal between transmembrane domain to ATP-binding domain [41]. Although the 10 µM concentration is high compared with the concentration of venetoclax used as a BCL-2 inhibitor, based on previous publications, this concentration is common among targeting cancer treatment drugs as reversal agents. For example, gefitinib effectively reverses ABCG2-mediated MDR at 10 µM, sildenafil reverses ABCB1- and ABCG2-mediated MDR at 10 µM, ciprofloxacin reverses ABCB1-mediated MDR at 10 µM, tivozanib reverses ABCB1- and ABCG2-mediated MDR at 5 µM [45,46,47,48]. In conclusion, venetoclax at a relatively high concentration (10 µM) antagonized wild-type ABCG2-mediated MDR by increasing drug intracellular accumulation as a result of the drug efflux function.

## 4. Materials and Methods

### 4.1. Chemicals

Anticancer drugs and positive reversal agents: Venetoclax was purchased from ChemieTek (Indianapolis, IN, USA). Paclitaxel, mitoxantrone, topotecan, SN-38, verapamil, KO143, and cisplatin were purchased from Sigma Co (St. Louis, MO, USA). Cell culture: Bovine serum (BS), fetal bovine serum (FBS), Dulbecco’s modified eagle’s medium (DMEM) and trypsin (0.25%) were purchased from Corning Inc. (Corning, NY, USA). Cell viability assay: dimethylsulfoxide (DMSO), 3-(4,5-dimethylthiazol-2-yl)-2,5-diphenyltetrazolium bromide (MTT) were purchased from Sigma Co. Immunoblotting: Human monoclonal antibodies for ABCG2 and GAPDH were purchased from Millipore (Billerica, MA, USA). Alexa Fluor 488 conjugated secondary antibody was purchased from Thermo Fisher Scientific Inc (Rockford, IL, USA). Triton X-100, 4′,6-diamidino-2-phenylindole (DAPI) were purchased from Sigma Co. Other chemicals: [^3^H]-labeled mitoxantrone (2.5 Ci/mmol) was purchased from Moravek Biochemicals (Brea, CA, USA). All other chemicals were purchased from Sigma Co.

### 4.2. Cell Lines

Human embryonic kidney cell line HEK293, transfected with empty or ABCG2-recombinant vector, were used as single-factor resistant controls in this study. Specifically, pcDNA3.1 (empty vector), pcDNA3.1-ABCG2-R482 (full-length wild-type ABCG2), pcDNA3.1-ABCG2-R482G (full-length mutated ABCG2 with glycine at position 482) or pcDNA3.1-ABCG2-R482T (full-length mutated ABCG2 with threonine at position 482) were used to transfect HEK293 cells to create parental or ABCG2-overexpressing cells. All HEK293 transfected cell lines were cultured in DMEM (10% FBS, 1% P/S) containing 2 mg/mL G418 at 37 °C in a humidified environment with 5% CO_2_. The non-small cell lung cancer (NSCLC) cell line NCI-H460, human colon adenocarcinoma cell line S1, and human epidermoid carcinoma cell line KB-3-1 were used in this study. Meanwhile, NCI-H460/MX20 and S1-M1-80 cells, selected from NCI-H460 and S1 cells, respectively, by mitoxantrone, as well as KB-C2, selected from KB-3-1 cells by colchicine were also used in this study. These pairs of drug-selected cell lines were chosen to verify the antagonistic effect of venetoclax on wild-type ABCG2 and mutated ABCG2. Both NCI-H460/MX20 and S1-M1-80 cells are ABCG2-overexpressing MDR cancer cells. DNA sequencing revealed that NCI-H460/MX20 cells express wild-type ABCG2 (R482) and S1-M1-80 cells express mutated ABCG2 (R482G) [24]. KB-C2 is an MDR cell line overexpressing ABCB1. All resistant cell lines were cultured and maintained as previously described [49]. All resistant cells were cultured in drug-free medium at least 2 weeks before the experiment.

### 4.3. MTT-Based Cytotoxicity Assay

Venetoclax cytotoxicity and reversal effects were measured based on modified MTT colorimetric assay as previously described [50]. In this study, the reversal effect was determined by the variation in the IC_50_ of chemotherapeutic agents. Specifically, cells were seeded equally into 96-well plates at a final concentration of 4000–6000 cells/well. After one night, 0.5 to 10 µM venetoclax or a positive control of an MDR modulator was added 2 h prior to adding gradient concentrations (0–30 µM) of an anti-cancer drug. Plates were cultured in the same environment as described in Section 4.2 for 68 h, after which the MTT solution was added to each well at a final concentration of 0.4 mg/mL and cultured for additional 4 h at 37 °C. Subsequently, the medium was discarded, 100 µL DMSO was added to each well, and the plate was shaken until all formazan was dissolved. Absorbance at 570 nm was measured by a microplate reader (Dynex Technology, VA, USA). Cell viability was calculated by setting the viability of the untreated (or 0 µM) cells as 100%. IC_50_ values were calculated from the concentration-viability curve by a modified Bliss method, as previously described [51]. In the reversal study, verapamil and KO143 were used as the ABCB1 and ABCG2 positive modulator, respectively.

### 4.4. Western Blotting

The Western blotting protocol has been described in a previous publication [52] with minor modifications. Cells were incubated with or without venetoclax for 0, 24, 48, or 72 h. Corresponding cells were lysed at each time point and lysates were stocked at −80 °C before the protein amount was quantified using BCA protein assay kit (Pierce, IL, USA). For protein immunoblotting assay, an equal amount of protein (25 µg) was loaded into a 10% sodium dodecyl sulfate–polyacrylamide gel (SDS-PAGE) and transferred to polyvinylidene difluoride (PVDF) membranes for antibody binding. Primary antibody (1:1000) and secondary antibody labelled with HRP (1:2000) were used to determine protein (ABCG2 and GAPDH) presence. Protein bands were visualized by a C-Digit Blot Scanner (LI-COR, NE) and analyzed by Image J software.

### 4.5. Mitoxantrone[^3^H] Accumulation Assay

In this study, the accumulation assay was performed as previously described, with minor modification [53]. Cells were seeded equally into 24-well plates at a density of 100,000 cells/well and cultured overnight. Then, cells were incubated with venetoclax for 2 h before incubation in a medium containing mitoxantrone[^3^H] for another 2 h. Subsequently, the medium was removed and the cells were rinsed with PBS, then the cells were collected and lysed. Lysates were transferred into equal amounts of scintillation fluid, after which radioactivity was determined in a liquid scintillation analyzer (Packard Instrument, IL, USA).

### 4.6. Mitoxantrone[^3^H] Efflux Assay

An efflux assay was performed following a modified protocol of the accumulation assay [54]. Specifically, cells were lysed after incubation with venetoclax (2 h), venetoclax + mitoxantrone[^3^H] and venetoclax (2 h). Then the cells were rinsed with PBS and lysed at different time points (0, 0.5, 1, or 2 h). Radioactivity was measured as described in Section 4.5.

### 4.7. ATPase Assay

Vanadate-sensitive ABCG2 ATPase activity was determined as previously described [55]. In brief, an equal amount (10 µg) of wild-type ABCG2-overexpressing cell membranes purchased from BD Biosciences (San Jose, CA, USA) were incubated with an assay buffer containing 5 mM sodium azide (NaN_3_), 1 mM ouabain (g-strophanthin), 2 mM dithiothreitol (DTT), 10 mM magnesium chloride (MgCl_2_), 50 mM potassium chloride (KCl), 2 mM ethylene glycol-bis(β-aminoethyl ether)-N,N,N′,N′-tetra acetic acid (EGTA) and 50 mM pH 6.8 2-(N-morpholino) ethanesulfonic acid (MES), with or without 0.3 mM sodium orthovanadate (Na_3_VO_4_), at 37 °C for 5 min. Then the mixture was incubated with 0–40 µM venetoclax at the same temperature for 3 min. Mg-ATP (5 mM) solution was then added to initiate a 20 min reaction at 37 °C, followed by adding 5% SDS to terminate the reaction. The amount of inorganic phosphates was determined by a colorimetric method, as previously described [56].

### 4.8. Molecular Docking of Venetoclax with Wild-Type Human ABCG2 Model

The venetoclax 3-D structure was constructed for docking simulation with a human ABCG2 model as previously described [57,58]. Wild-type human ABCG2 protein model (6ETI) was obtained from RCSB Protein Data Bank (RCSB PDB, http://www.rcsb.org). The protein structure was an inward-facing inhibitor-bound wild-type (R482) ABCG2 with a resolution of 3.1 Å [12]. Docking calculations were performed in AutoDock Vina (version 1.1.2) [59]. Hydrogen atoms and partial charges were added using AutoDockTools (ADT, version 1.5.4). Docking conformation and interactions were visualized in PyMOL (version 2.3, educational version). Docking grid center coordinates were determined from the bound inhibitor in 6ETI model. When docking within the ATP-binding site, the grid center was set referring to another ATP-binding human ABCG2 model (6HZM) by locating the same sets of residues surrounding the bound ATP. Receptor/ligand preparation and docking simulation were performed using default settings. The top-scoring (affinity score: kcal/mol) result was selected for further analysis and visualization. All docking computations were performed on a MacBook Pro with 4-core CPU and macOS Mojave 10.14.3 system.

### 4.9. Molecular Dynamics (MD) Simulation

The best-scored docked complex generated in Section 4.8 was subjected to molecular dynamics simulation for binding stability validation. The simulation was performed in Desmond, a free software for non-commercial users, developed by DE Shaw Research Group [60]. The protein-membrane-ligand-solvent system was established in the Maestro Module System Builder with POPC bilayer membrane, TIP3P water and counter ions to neutralize the charge. The system was equilibrated by the Desmond default protocol while other parameters were kept in the default setting (constant temperature 300 K, 1.015 bar pressure). The complex system was subjected to 50,000 ps (50 ns) simulation. MD computations and visualizations were performed in the Ubuntu 16.04 system with 16-core CPU, 60 GB memory and an NVIDIA^®^ (Santa Clara, CA, US) Tesla P100 GPU.

### 4.10. Statistics

The results in this study are presented as mean ± SD and analyzed using one-way ANOVA. All data was generated from at least three independent experiments with triplicates or duplicates.

## 5. Conclusions

Our study unveiled the mechanism of synergy between venetoclax, an FDA-approved BCL-2 inhibitor, and chemotherapeutic agents in MDR cells overexpressing wild type ABCG2. The mechanism lies in blocking the efflux function of wild type ABCG2 transporter by directly binding to the drug-binding pocket as well as the ATP-binding site, without altering the ABCG2 expression. Our findings provide evidence that venetoclax in combination therapy may be a beneficial strategy to overcome wild-type ABCG2-mediated cancer MDR.

## Supplementary Materials

The following are available online at https://www.mdpi.com/2072-6694/12/2/466/s1, Figure S1: The effect of venetoclax in drug-selected ABCG2-overexpressing cells, Figure S2: Whole blotting of H460 + H460/MX20, Figure S3: Whole blotting of S1 + S1-M1-80, Figure S4: Whole blotting of HEK293/pcDNA3.1 and HEK293/ABCG2 (wild-type and mutants).
