# Supplementary material for: Venetoclax, a BCL-2 Inhibitor, Enhances the Efficacy of Chemotherapeutic Agents in Wild-Type ABCG2-Overexpression-Mediated MDR Cancer Cells
Source: Cancers (Basel). 2020 Feb 18;12(2):466. doi: 10.3390/cancers12020466 (PMC7072352; doi:10.3390/cancers12020466)
Supplement: Supplementary file 1 [file cancers-12-00466-s001.pdf]

cancers

MDPI

Supplementary Materials:

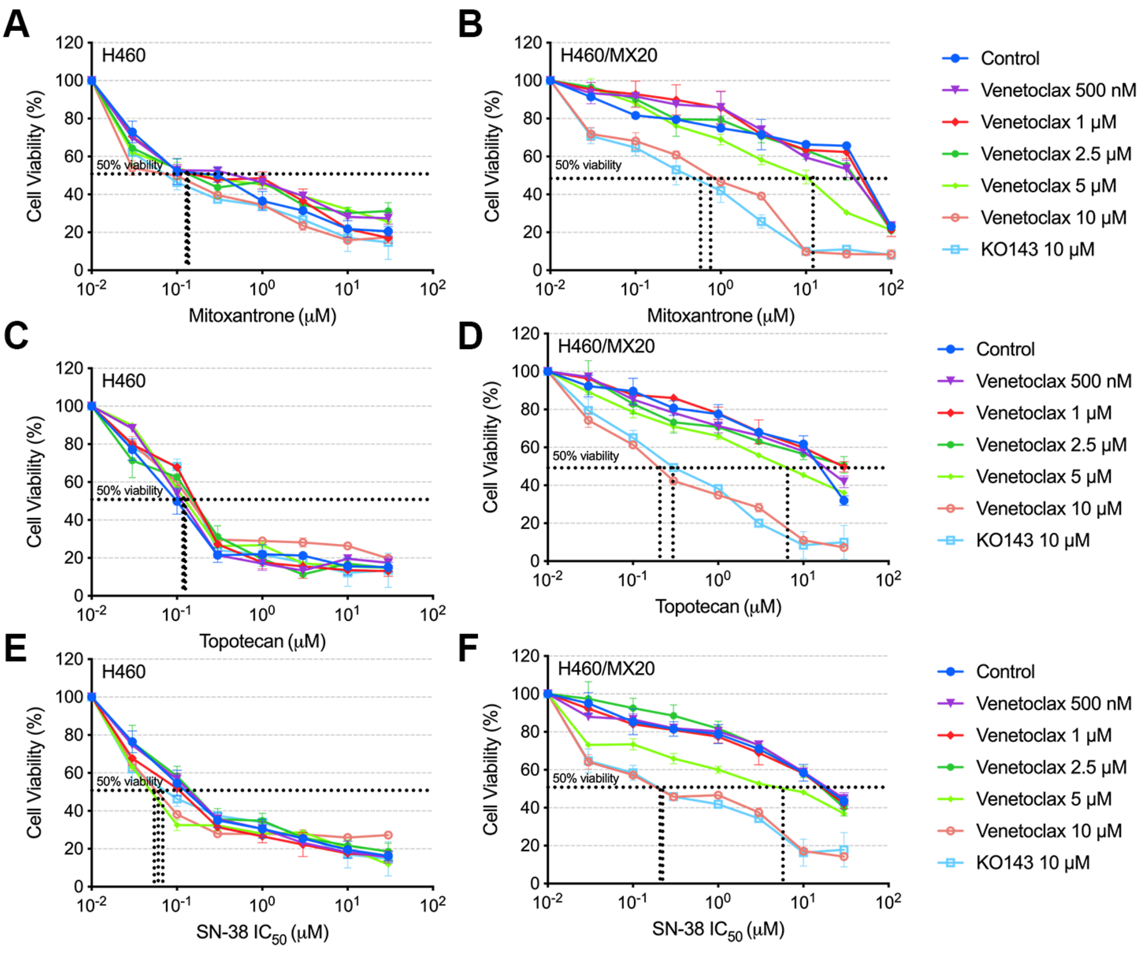

Figure S1. The effect of venetoclax in drug-selected ABCG2-overexpressing cells. Cell viability of (**A**) H460 or (**B**) H460/MX20 incubated for 72 h with mitoxantrone, mitoxantrone + venetoclax and mitoxantrone + KO143. (**C**) H460 or (**D**) H460/MX20 incubated with topotecan, topotecan + venetoclax and topotecan + KO143. (**E**) H460 or (**F**) H460/MX20 incubated for 72 h with SN-38, SN-38 + venetoclax and Sn-38 + KO143. Black dash lines indicate 50% cell viability (horizontal) and corresponding IC_{50} values of anticancer drugs (vertical), anticancer drugs + 5/10 μM venetoclax or 10 μM KO143.

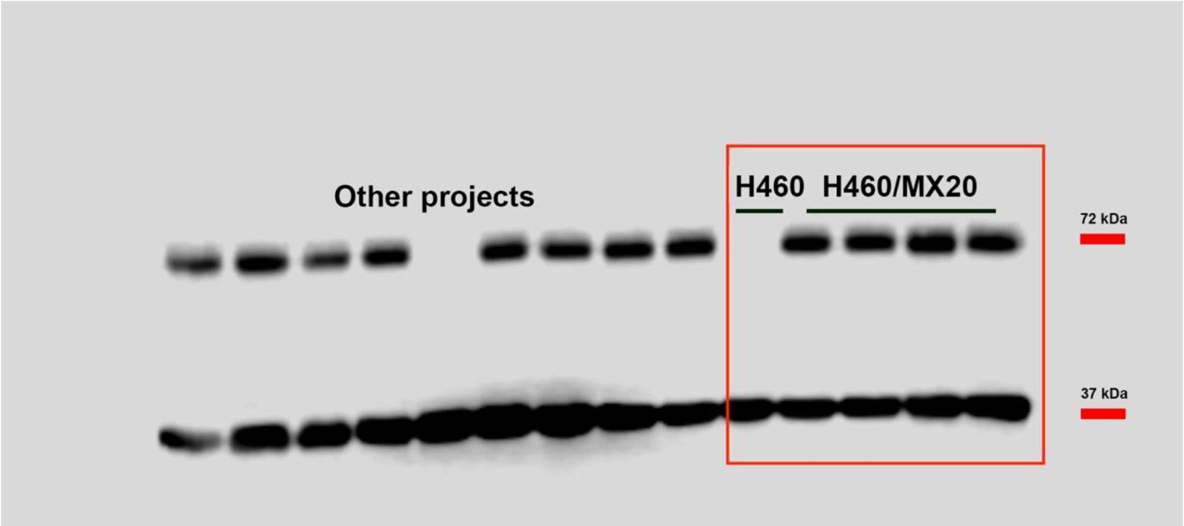

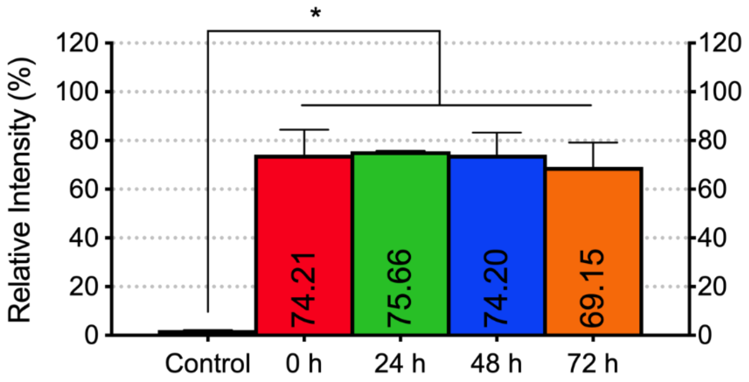

Figure S2. Whole blotting of H460 + H460/MX20. Bands circled by the red rectangular were H460 (left 1) and H460/MX20 (left 2–5). Molecular weight was marked by red rectangulars on the right side. Expression level quantification with relative grey scale values was displayed as bar graph.

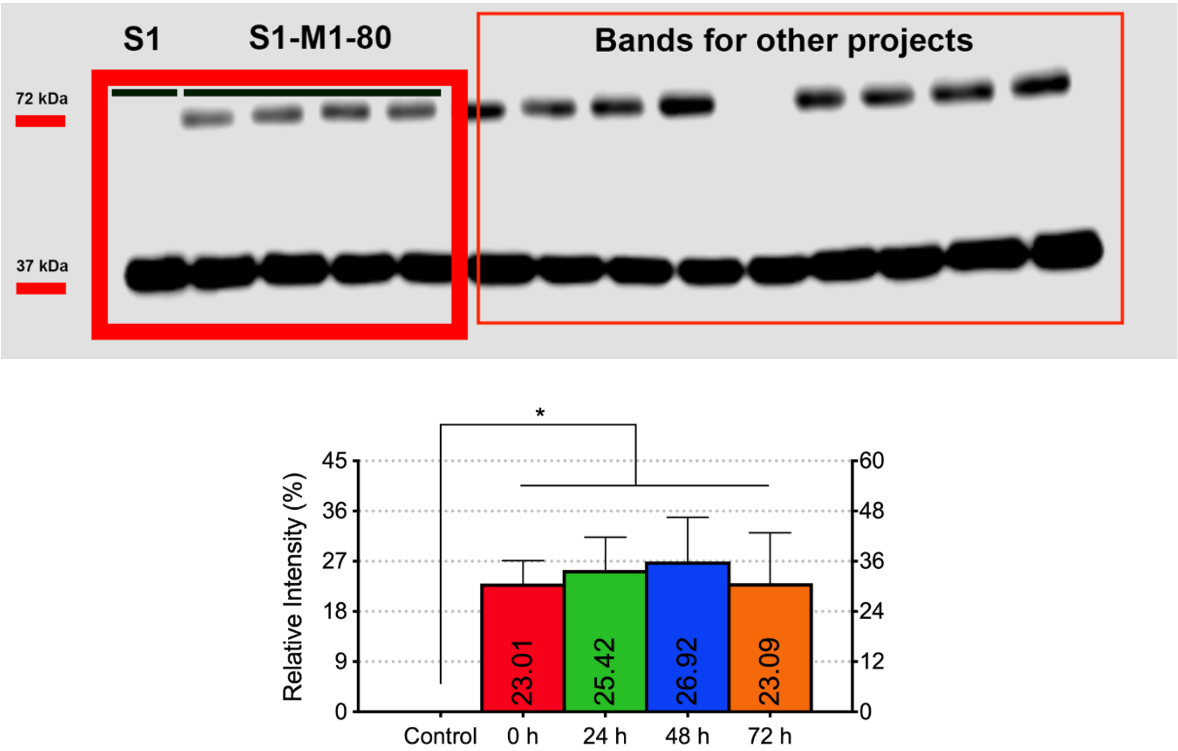

Figure S3. Whole blotting of S1 + S1-M1-80. Bands circled by the red rectangular were S1 (left 1) and S1-M1-80 (left 2–5). Molecular weight was marked by red rectangular on the left side. Expression level quantification with relative grey scale values was displayed as bar graph.

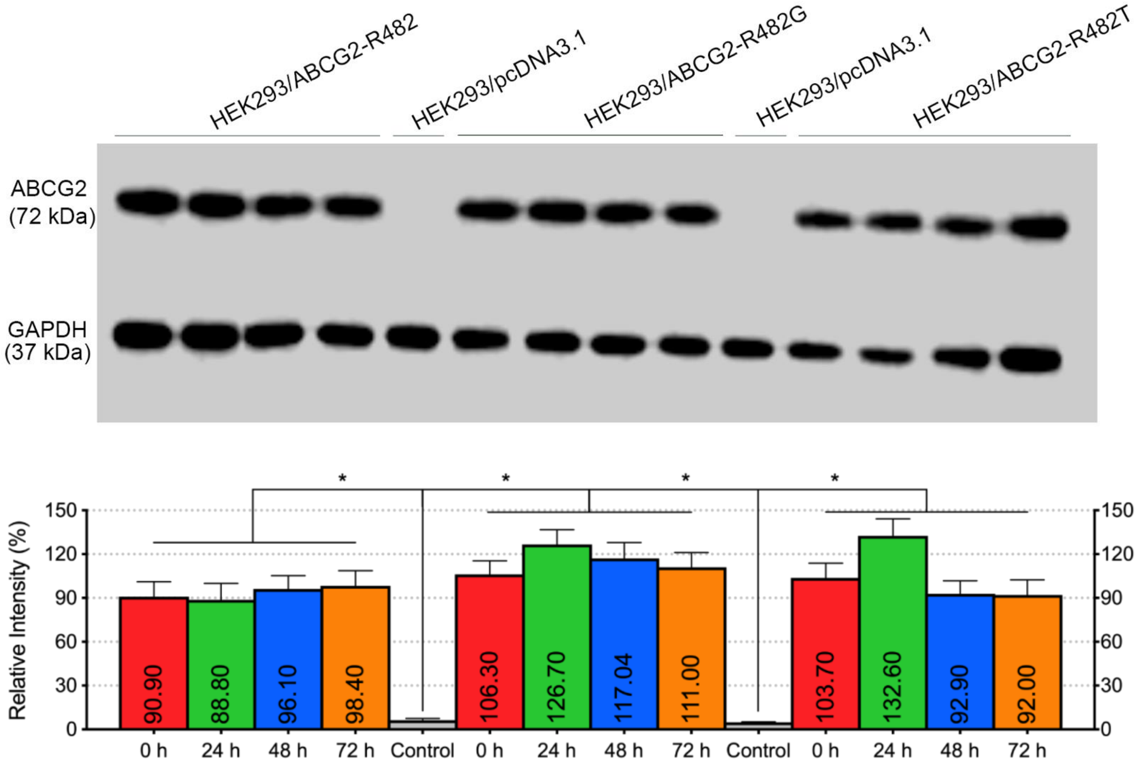

Figure S4. Whole blotting of HEK293/pcDNA3.1 and HEK293/ABCG2 (wild-type and mutants). Molecular weights were marked on the left side. Expression level quantification with relative grey scale values was displayed as bar graph.
